# Compatible bacterial mixture, tolerant to desiccation, improves maize plant growth

**DOI:** 10.1371/journal.pone.0187913

**Published:** 2017-11-08

**Authors:** Dalia Molina-Romero, Antonino Baez, Verónica Quintero-Hernández, Miguel Castañeda-Lucio, Luis Ernesto Fuentes-Ramírez, María del Rocío Bustillos-Cristales, Osvaldo Rodríguez-Andrade, Yolanda Elizabeth Morales-García, Antonio Munive, Jesús Muñoz-Rojas

**Affiliations:** 1 Laboratorio de Ecología Molecular Microbiana (LEMM), Centro de Investigaciones en Ciencias Microbiológicas (CICM), Instituto de Ciencias (IC), Benemérita Universidad Autónoma de Puebla (BUAP), Edificio IC11, Ciudad Universitaria, Colonia Jardines de San Manuel, Puebla, Puebla, México; 2 Laboratorio de Biología Molecular y Microbiología, Facultad de Ciencias Biológicas, BUAP, Edificio 112-A, Ciudad Universitaria, Colonia Jardines de San Manuel, Puebla, Puebla, México; 3 CONACYT, LEMM, CICM, IC-BUAP, Edificio IC11, Ciudad Universitaria, Colonia Jardines de San Manuel, Puebla, Puebla, México; 4 Genética Molecular Microbiana, CICM, IC-BUAP, Edificio IC11, Ciudad Universitaria, Colonia Jardines de San Manuel, Puebla, Puebla, México; Dong-A University, REPUBLIC OF KOREA

## Abstract

Plant growth-promoting rhizobacteria (PGPR) increase plant growth and crop productivity. The inoculation of plants with a bacterial mixture (consortium) apparently provides greater benefits to plant growth than inoculation with a single bacterial strain. In the present work, a bacterial consortium was formulated containing four compatible and desiccation-tolerant strains with potential as PGPR. The formulation had one moderately (*Pseudomonas putida* KT2440) and three highly desiccation-tolerant (*Sphingomonas* sp. OF178, *Azospirillum brasilense* Sp7 and *Acinetobacter* sp. EMM02) strains. The four bacterial strains were able to adhere to seeds and colonize the rhizosphere of plants when applied in both mono-inoculation and multi-inoculation treatments, showing that they can also coexist without antagonistic effects in association with plants. The effects of the bacterial consortium on the growth of blue maize were evaluated. Seeds inoculated with either individual bacterial strains or the bacterial consortium were subjected to two experimental conditions before sowing: normal hydration or desiccation. In general, inoculation with the bacterial consortium increased the shoot and root dry weight, plant height and plant diameter compared to the non-inoculated control or mono-inoculation treatments. The bacterial consortium formulated in this work had greater benefits for blue maize plants even when the inoculated seeds underwent desiccation stress before germination, making this formulation attractive for future field applications.

## Introduction

The inoculation of plants with beneficial microorganisms is a practice used in modern agriculture and offers advantages to crops, such as increasing plant growth and triggering protection to different diseases [1,2]. Plant growth-promoting rhizobacteria (PGPR) are bacteria influenced by plant root exudates that have the ability to improve plant growth over the short term [3] and crop production over the long term [4,5]. Some examples of PGPR include strains of *Azospirillum brasilense*, *Bacillus subtilis*, *Bacillus amyloliquefaciens*, *Bradyrhizobium japonicum*, *Enterobacter cloacae*, *Gluconacetobacter diazotrophicus*, *Pantoea agglomerans*, *Pseudomonas putida*, *Pseudomonas fluorescens*, *Rhizobium leguminosarum*, and *Sinorhizobium meliloti* [6,7].

There are several ways by which PGPR could directly facilitate plant growth, such as biological N_2_ fixation, phosphate solubilization and phytohormone production [3,8]. In addition, PGPR could indirectly promote plant growth by preventing the negative effects of phytopathogenic organisms through the production of antimicrobial compounds or the elicitation of induced systemic resistance (ISR) [7,9]. Sustainability issues in agriculture can no longer be overlooked and are a priority for several countries around the world [2,10]; in this regard, the application of microbial inoculants to the fields might help to ensure sustainable crop production at low cost.

The inoculation of seeds with multiple beneficial bacteria might have greater potential for plant growth promotion and biological control than inoculation with a single bacterial species [1,11]. Bacterial consortiums as inoculants could have superior effects on plants since different types of microorganisms might interact synergistically to provide nutrients, remove inhibitory products and stimulate one another through physical or biochemical activities that may enhance some beneficial aspects of their physiology [12,13]. Some examples include the co-inoculation of chickpea with *Serratia marcescens* (SF3), *Serratia* spp. (ST9), and *Mesorhizobium ciceri*, which increased the number of nodules per plant, nodule dry weight, number of pods per plant, grain yield, protein content, and total chlorophyll content under irrigated and rainfed conditions compared to inoculation with single bacterial strains [14]. Sugarcane inoculation with a consortium of 5 diazotrophic bacteria (*Gluconacetobacter diazotrophicus*, *Herbaspirillum seropedicae*, *Herbaspirillum rubrisubalbicans*, *Azospirillum amazonense*, and *Paraburkholderia tropica*) also showed higher stem production in two soils with low-to-medium levels of chemical fertilizer compared to mono-inoculated plants [15].

The design, formulation and optimization of effective bacterial consortium inoculants is not an easy task; it requires a deep understanding of modes of interaction, bacterial adhesion to seeds and plant root colonization [1,11]. Furthermore, antagonistic relationship studies should be conducted before the design and application of formulations containing bacterial consortiums since some antagonistic effects may occur in bacterial consortiums associated with plants [15].

Another issue impacting the effectiveness of PGPR is drought stress, which is particularly important when rhizobia are used as plant inoculants since desiccation affects cell viability during storage in carrier-based inoculants and field soils [16–18]. Therefore, desiccation-tolerant bacteria are highly desirable because they can remain in soils and inoculant formulations for a longer time than those that are not tolerant to desiccation, and they can improve plant colonization under low water availability [16,18]. In natural environments, organisms termed anhydrobionts are able to survive desiccation by entering a dormant stage during which metabolism is undetected [19]. When rehydrated, these organisms quickly restore their metabolic processes and resume life [20]. In the present work, four compatible bacterial strains tolerant to desiccation with potential as PGPR were selected to formulate a liquid bacterial consortium. Seeds of autochthonous blue maize (Papalotla, Tlaxcala, México) were inoculated with the bacterial consortium and the individual bacterial strains, and their ability to adhere, colonize and promote growth was evaluated in plants grown from inoculated seeds subjected to two experimental conditions before sowing: normal hydration (without desiccation stress) or desiccation (with desiccation stress). Maize is the most important cereal in Mexico and is preponderant in Latin America; furthermore, it serves as raw material for the production of processed food, such as snacks and cereal bars [21]. Autochthonous blue maize was selected in this work because of its importance in the preparation of traditional Mexican food [21] and the health benefits related to its high content of anthocyanin. Moreover, studies have documented that blue maize anthocyanin modifies the structure of starch, generating an antioxidant environment in the colon that prevents cellular proliferation and cancer [22,23].

## Materials and methods

### Bacterial strains

Twenty bacterial strains belonging to the genera *Azospirillum*, *Acinetobacter*, *Bradyrhizobium*, *Rhizobium*, *Burkholderia*, *Enterobacter*, *Pseudomonas*, *Paraburkholderia* and *Sphingomonas* were used in initial screening assays (S1 Table).

### GenBank accession number

The sequence of the gene encoding the 16S rRNA from *Acinetobacter* sp. EMM02 (partially characterized in this work) was deposited in the GenBank database with the accession number KU686485.

### Blue maize

Autochthonous blue maize was collected from a field located in San Diego Buena Vista, Papalotla de Xicohténcatl, Tlaxcala, México (Latitude: 19.172353, Longitude: -98.164799). This blue maize has been cultivated every year for a very long time in that location. Because the maize used in this work has not been previously described, we propose to denominate this maize blue maize CAP15-1 TLAX.

### Soil

The soil used in the present work was collected from the same locations where the blue maize was sampled. Permission to collect the soil was granted by Mrs. Iris Sauz Muñoz (owner of the land) and endorsed by the president of the Papalotla Tlaxcala Ejidal Committee.

### Antagonism assays

Twenty strains were tested both as producers of antagonistic substances and as indicator strains using the double agar layer method (dx.doi.org/10.17504/protocols.io.j4mcqu6) [24,25]. We used PY-Ca medium for these assays because this is a wide-spectrum growth medium, and all the strains used in this work were able to grow. PY-Ca contains 5 g peptone, 3 g yeast extract, and 0.7 g calcium chloride per liter of medium [26,27].

### Bacterial desiccation assays

Different strains that are able to co-exist with other strains were used to carry out desiccation assays as described in an established protocol (dx.doi.org/10.17504/protocols.io.j4icque) in SYP medium (10 g sucrose, 3 g yeast extract, 1 g K_2_HPO_4_, 3 g KHPO_4_, 1 L water, pH 6). Strains were grown until the stationary phase for the desiccation experiments because the bacterial tolerance to desiccation increases during this stage in comparison to the exponential phase [28]. The bacterial suspensions contained approximately 1 × 10^9^ CFU/mL before desiccation. The counting of cultivable cells was conducted after the rehydration of the desiccated cells every three days for a period of twelve days using the massive stamping drop plate (MSDP) method [29], and the bacterial survival ratio (BSR) was calculated as the ratio of the log of the number of bacterial cells present in the suspension at any time after the beginning of desiccation (AbD) plus one to the log of the number of viable cells before desiccation (BD), multiplied by 100, i.e., BSR = (log AbD+1/log BD) × 100 [24]. The BSR is a parameter that allowed us to quantify the desiccation tolerance of the bacteria.

### Compatibility of the strains selected for the bacterial consortium

Bacteria were grown individually or together in SYP and PY-Ca liquid medium at 30°C and 200 rpm for 120 hours; each culture was generated in triplicate. The number of cultivable cells was quantified in quintuplicate using the MSDP method with selective medium plates to identify any inhibitory relationships among the co-inoculated strains as described in other works [24,30].

For these experiments bacteria were quantified in their own selection medium, developed in this work, that allow the growth of one of the four strains and limit the growth of the others. We began the screening with reported media such as Congo Red [31], BAc [32], LB, and MM9 [28], and others. The growth of the strains was assayed in these media supplied with different antibiotics and concentrations of them. Media used for selection were BAc CTX^30^ (Cefotaxime 30 μg/ml) for *Acinetobacter* sp. EMM02, Congo Red Cro50 (Ceftriaxone 50 μg/ml) for *A*. *brasilense* Sp7, MM9- citrate Cm^150^ for *P*. *putida* KT2440, and Luria Bertani modified with only 5% of components AK^50^ (Amikacin 50 μg/ml).

### Desiccation tolerance of the strains selected for the bacterial consortium in different types of soils

Two types of substrate, sandy soil and loamy sandy soil, were used to perform desiccation experiments. The physicochemical characteristics of the soils were determined as in previous studies [14]. A bacterial suspension was obtained as indicated in the section “Bacterial desiccation assays”, and the number of cultivable cells was quantified in quintuplicate using the MSDP method with SYP medium plates. For each strain, thirty sterile Eppendorf tubes were filled with 500 mg of experimental soil plus 500 μL of the bacterial suspension; they were mixed using strong agitation and covered with a sterile cotton cap. Treatments composed of 500 μL of bacterial suspension without soil for support were included. All tubes were placed in a desiccation chamber at 30°C and 55% Relative Humidity (RH). The water in each tube was completely evaporated 5 days after placing the tubes in the desiccation chamber. The counting of cultivable cells was performed every three days for a period of twelve days using the MSDP method. For the samples of 3 DABD, five tubes were used for counting, adjusting the liquid volume of each tube to the initial 500 μL. For the completely desiccated samples (6, 9, and 12 DABD), 500 μL of water was added to the cells, and after 40 min of occasional shaking, rehydration was achieved.

### Determination of the PGPR characteristics of the bacterial consortium

Phosphate solubilization was done in Pikovskaya broth medium supplemented with broth containing 5 g/L of Ca_3_ (PO_4_)_2_ after 5 days of culture growth at 30°C [33,34].

Indole compounds were estimated using the colorimetric assay based on the Salkowski reagent and using the PC reagent and K-lactate medium supplemented with tryptophan (100 ppm) [14,35].

The siderophore content was determined according to the fast and universal method involving chrome azurol S (CAS) with an overlay technique in which a modified O-CAS medium is layered over culture agar plates [36]. The bacteria were grown on LB agar plates for 48 h at 30°C, and then one layer was treated with 30 mL of O-CAS medium without nutrients and incubated at 30°C for 15 min. Subsequently, change in color from blue to orange was observed around the growth of the siderophore-producing bacterial strains.

### Seed inoculation procedure

Bacterial cells were grown in PY-Ca medium until the stationary phase was attained; the cells were harvested using centrifugation, washed twice and resuspended in sterile water at the same initial volume (100 mL). The bacterial consortium was formulated with 25 mL of each washed bacterial suspension. The number of cells was determined in quintuplicate using the MSDP method with selective solid media (S3 Table). The seeds were washed with sterile water, rinsed with 70% ethanol for 10 min, immersed in 6.5% sodium hypochlorite, and agitated for 20 min; afterward, the seeds were washed eight times under sterile conditions [37]. One hundred thirty-five seeds of blue maize were soaked in each bacterial suspension (*A*. *brasilense* Sp7, *P*. *putida* KT2440, *Acinetobacter* sp. EMM02, *Sphingomonas* sp. OF178 and the bacterial consortium) for 60 min. Non-inoculated seeds were included as a control, and they were soaked in distilled sterile water for 60 min. The inoculated seeds were subjected to two experimental conditions before sowing: without desiccation stress (Exp. 1) or with desiccation stress (Exp. 2), following which seeds from both treatments were used in growth promotion assays.

#### Experiment 1. Inoculated seeds without desiccation stress

Fifty seeds from each inoculation treatment were sown in pots containing 650 g of sterile vermiculite. Five hundred milliliters of sterile liquid MS [38] and 200 mL of water were added to each pot. All pots were placed in a greenhouse under 16 hours of light and a temperature of 30°C during the daytime and 8 hours of dark and a temperature of 25°C during the night. The plants were grown for 45 days and watered regularly with distilled water.

#### Experiment 2. Inoculated seeds with desiccation stress

Eighty-five seeds from each inoculation treatment were placed into Petri dishes containing dry sterile filter paper (Whatman No. 1). The dishes containing seeds were placed in a desiccation chamber at 30°C, 55% RH. Water was completely lost at 1 DABD under these conditions. Every three days after the beginning of desiccation, five seeds were removed from the desiccation chamber. Each one was placed in a 15 mL capacity tube containing 3 mL of sterile water for 2 h, and the tubes were vigorously agitated using a vortex. The number of cultivable bacteria was quantified using the MSDP method, and the BSR was calculated. Eighteen days after the beginning of desiccation (DABD), 50 inoculated seeds from each treatment and 50 non-inoculated seeds were sown in pots containing 650 g of sterile vermiculite and supplemented with 500 mL of sterile liquid MS medium [4,38] and 200 mL of water. Under these conditions, the bacteria associated with the seeds were rehydrated. The pots of all treatments were placed in a greenhouse with 16 hours of light and a temperature of 30°C during the daytime and 8 hours of dark and a temperature of 25°C during the night. The plants were grown for 45 days and watered regularly with distilled water.

### Adherence and colonization assays

The bacterial cells that had adhered to the seed surfaces were quantified 12 h after being sown in vermiculite for both experiments 1 and 2; five replicates of inoculated seeds from each treatment were extracted from the pots containing vermiculite and vigorously vortexed in 3 mL of sterile water [4]. Each generated suspension was used to determine the bacterial number using the MSDP method with agar plates containing selective media [29].

Rhizospheric colonization was evaluated for all treatments of experiments 1 and 2. Briefly, five plants from each treatment were harvested from the pots at fifteen, thirty and forty-five days after inoculation. The roots were shaken to discard non-adhered vermiculite. The roots of each plant with strongly adhered vermiculite were immersed in enough sterile water to cover the roots and shaken vigorously. The bacterial suspension was used to quantify the rhizospheric bacteria using the MSDP method. The weight of the vermiculite was obtained by drying samples without the roots as previously described [4]. Bacteria were inoculated onto selective media plates for the quantification of each experimental species, and incubation was carried out for 24 h at 30°C [37].

### Measurement of plant growth parameters

After 45 days, the plants were removed from the vermiculite and washed with water, and the excess water was dried using absorbent paper. The shoot was measured using a tape measure to obtain the diameter and plant height. The fresh weight of the seedlings was determined with the help of an analytical balance; thereafter, the samples were oven dried at 75°C to a constant dry weight, and the dry weight data were recorded. The same procedure was used for both experiments.

### Genomic DNA extraction, amplification of genes encoding 16S rRNA and restriction patterns

The DNA of the strains was extracted using a genomic DNA purification kit (PROMEGA).

From each sample of extracted DNA, the gene encoding 16S rRNA was amplified using PCR with the conserved primers fD1: 5´-AGAGTTTGATCCTGGCTCAG-3´ and rD1: 5´-AAGGAGGTGATCCAGCC-3´ [39], which amplify almost the full length of the 16S rRNA gene (1500 pb), and the Master MIX reagent (Invitrogen). Amplification was performed as described in [4]. PCR amplification was verified by agarose gel electrophoresis, and the amplified genes were purified using a PCR purification kit (Qiagen) according to the manufacturer´s instructions.

For each reference strain and strain isolated from the rhizosphere, the amplified gene encoding 16S rRNA was digested with 5 U of different restriction enzymes (*Alu*I, *Hha*I, *Hinf*I, *Rsa*I, *Mbo*I and *Msp*I). The lengths of the restriction fragments generated for each tested strain were determined by electrophoresis in 2% agarose gels using a commercial molecular weight marker (100 bp DNA ladder), and the patterns of the isolated strains were compared with those of initially inoculated reference strains [4,37,40].

### Statistical analysis

The statistical analysis of desiccation tolerance, bacterial consortium compatibility, and plant growth parameters was performed using SigmaPlot (Handel Scientific Software). Differences were evaluated according to Student’s *t* and Tukey’s tests. The results of the Student’s *t*-test comparison were used to generate a matrix of differences and similarities between treatments for the assignment of letters. For Tukey’s test, the data were analyzed globally by one way ANOVA, and comparisons were carried out with Tukey’s test.

## Results and discussion

The design, formulation and optimization of effective bacterial consortiums as inoculants require studies of co-interaction among members of the consortium, bacterial adhesion to seeds, plant root colonization and the ability of the bacteria to promote plant growth [1,11,41]. However, some environmental factors could affect the effectiveness of an inoculant, such as soil type [42,43], the variety and physiology of plants [40,43,44], salinity [40], water availability [17,40] and others. Bacterial coexistence is essential for the formulation of stable bacterial consortiums [15]. Bacteria may coexist if they do not produce inhibitory substances against one another, but some strains that produce such inhibitory substances could still coexist with resistant strains.

### Antagonism assays for the selection of compatible bacteria

In the present work, twenty bacterial strains belonging to the genera *Azospirillum*, *Acinetobacter*, *Bradyrhizobium*, *Rhizobium*, *Burkholderia*, *Enterobacter*, *Paraburkholderia*, *Pseudomonas*, and *Sphingomonas* (S1 Table) were used to evaluate antagonistic activity. They were tested as producers of antagonistic substances and as indicator strains (Table 1). *Rhizobium* sp. MS24 and *Bradyrhizobium* sp. MS13 were inhibited by 5 and 7 of the tested bacteria, respectively. In contrast, *Sphingomonas* sp. DS204 showed a broad spectrum of inhibition toward the majority of the tested bacteria. Thus, those bacteria were not considered in further experiments because of their high sensitivity or strong inhibitory effect, as such characteristics make them incompatible and unable to work together in a microbial consortium. Among the other seventeen strains, 11 could be able to coexist since they did not show antagonistic effects on the growth of the examined bacterial strains (Table 1). In addition, *Acinetobacter* sp. EMM02, *P*. *putida* KT2440, *Sphingomonas* sp. GOF-203, *Sphingomonas* sp. DS-201, *P*. *putida* DOT-T1E, and *Enterobacter* sp. UAPSO3001 showed antagonistic effects toward some strains, but they could still be compatible with the majority of bacterial strains considered in this work (Table 1). Therefore, we consider these 17 strains with the potential of being part of specific bacterial mixtures because they could coexist; considering their inhibitory relationship.

**Table 1:** Antagonism assays using the double agar layer method.

	Bacterial strains explored as sensitive																			
Bacterial strains explored as producer	1	2	3	4	5	6	7	8	9	10	11	12	13	14	15	16	17	18	19	20
*Acinetobacter* sp. EMM02			**P**				**P**	**P**	**P**			**P**				**P**				
*Azospirillum brasilense* Sp7																				
*Bradyrhizobium* sp. MS13																				
*Bradyrhizobium* sp. MS21																				
*Bradyrhizobium* sp. MS22																				
*Bradyrhizobium* sp. MS23																				
*Enterobacter* sp. UAPSO3001		**P**	**P**													**P**				
*Paraburkholderia tropica* MOc-725																				
*Paraburkholderia tropica* MTo-293																				
*Paraburkholderia tropica* MTo-672																				
*Paraburkholderia tropica* Pp8^T^																				
*Paraburkholderia unamae* MTI-64I^T^																				
*Paraburkholderia unamae* ScCu 23																				
*Pseudomonas putida* DOT-T1E			**P**													**P**				
*Pseudomonas putida* KT2440			**P**				**P**	**P**	**P**			**P**				**P**				
*Rhizobium* sp. MS24																				
*Sphingomonas* sp. DS-201			**P**													**P**				
*Sphingomonas* sp. DS-204	**P**	**P**	**P**	**P**	**P**	**P**	**P**	**P**		**P**	**P**	**P**		**P**	**P**	**P**	**P**		**P**	
*Sphingomonas* sp. GOF-203			**P**													**P**				
*Sphingomonas* sp. OF-178A																				

**P** means that producer strain inhibited the growth of the strain tested as sensitive (designated with the corresponding number listed in the column). Empty boxes mean that the strain tested as sensitive was not inhibited by the strain explored as posible producer.

### Bacterial tolerance to desiccation

A lack of water availability in the field decreases the survival of bacteria on inoculated seeds, and this is an important limitation in terms of their positive effects on plant growth promotion [17,40]. In fact, desiccation-tolerant bacteria are able to promote the growth of plants under adverse conditions, as has been reported for *Pseudomonas* spp. and *Viridibacillus arenosi* IHB B7171 after the inoculation of seeds of maize and seedlings of tea, peas and wheat [45]. Other desiccation-tolerant bacteria, such as *Microbacterium* sp. 3J1 and *Arthrobacter koreensis* 5J12A, promote the growth of pepper, showing a correlation between the degree of tolerance to desiccation and the level of protection to drought that they provide to the plant [46].

Seventeen compatible strains that were previously selected as described earlier in this work were used to perform desiccation experiments (30°C, 50% HR). The bacterial strains exhibited different levels of tolerance to desiccation stress 12 days after the beginning of desiccation (DABD) (Table 2). Our results show that some strains were highly tolerant (i.e., *A*. *brasilense* Sp7, *Acinetobacter* sp. EMM02, and *Sphingomonas* sp. OF178), others were moderately tolerant (i.e., *Sphingomonas* sp. GOF203, *P*. *putida* KT2440 and *Bradyrhizobium* sp MS23), and some strains were only slightly tolerant (i.e., *P*. *unamae* ScCu23) (Table 2). Only three strains were highly sensitive to desiccation (*P*. *tropica* MTo-672, *P*. *tropica* MOc-725, and *P*. *unamae* ScCu23), and they were not included in further studies. Other studies have also shown that PGPR strains can tolerate desiccation at different levels, for example, *Gluconacetobacter diazotrophicus* PAl 5 R can resist 2 days of desiccation [47], while *Bradyrhzobium japonicum* tolerates 3 days of desiccation [48,49]. The ability to tolerate desiccation has been reported for other PGPR, such as *Sinorhizomium meliloti*, *Rhizobium etli*, *Rhizobium leguminosarum* and *B*. *japonicum* [17,50].

**Table 2:** Bacterial survival ratio (BSR) of bacterial strains after desiccation stress.

Strain	0 DABD	3 DABD	6 DABD	9 DABD	12 DABD
***Acinetobacter* sp. EMM02**	**100**	**99.6±1.7 a**	**98.9±1.1 a**	**97.2±1.2 a**	**92.4±0.7 a**
***Azospirillum brasilense* Sp7**	**100**	**99.0±1.2 a**	**96.5±1.9 a**	**95.5±0.8 b**	**90.0±1.1 b**
*Bradyrhizobium* sp. MS21	100	99.0±0.8 a	94.1±1.9 b	91.1±0.9 b	86.1±1.6 c
*Bradyrhizobium* sp MS22	100	95.2±2.2 b	88.4±1.9 c	87.6±2.3 c	85.7±0.6 c
*Bradyrhizobium* sp. MS23	100	86.2±1.0 b	85.4±1.9 c	77.8±0.5 e	74.8±1.0 e
*Enterobacter* sp. UAPSO3001	100	97.0±1.8 b	95.0±1.9 b	94.1±0.8 b	93.5±0.8 a
*Paraburkholderia tropica* MOc-725	100	76.0±2.4 d	50.0±1.2 f	ND	ND
*Paraburkholderia tropica* MTo-293	100	99.4±0.6 a	88.2±1.9 c	86.6±1.0 c	83.2±0.8 d
*Paraburkholderia tropica* MTo-672	100	93.1±1.8 b	61.0±2.3 e	ND	ND
*Paraburkholderia tropica* Pp8T	100	91.6±2.4 b	93.1±1.9 b	88.7±2.4 c	86.7±1.4 c
*Paraburkholderia unamae* MTl-641^T^	100	96.5±0.5 b	89.0±1.9 c	87.3±2.4 c	82.0±1.1 d
*Paraburkholderia unamae* ScCu23	100	81.4±2.5 c	60.0±2.2 e	ND	ND
*Pseudomonas putida* DOT-T1E	100	99.5±0.9 a	99.1±1.1 a	97.7±1.0 a	82.0±1.8 d
***Pseudomonas putida* KT2440**	**100**	**87.3±1.7 c b**	**83.1±0.7 d**	**73.5±0.9 e**	**60.0±0.9 f**
*Sphingomonas* sp. DS-201	100	99.3±1.1 a	97.3±1.9 a	96.0±2.0 a	85.7±1.1 c
*Sphingomonas* sp. GOF-203	100	88.2±1.3 b	86.6±1.9 c	82.3±1.6 d	74.7±0.9 e
***Sphingomonas* sp. OF-178A**	**100**	**97.0±0.9 b**	**92.8±1.9 b**	**91.8±1.7 b**	**89.6±0.8 b**

DABD means days after the beginning of desiccation. Values represent the media of five independent determinations and the respective standard deviation. Values with identical letters in the same column are not significantly different at p ≤ 0.05, as determined using Student’s *t* and Tukey’s tests. ND means that bacterial growth was not detected. Bold letters indicate the bacterial strains chosen for the bacterial consortium formulation.

### Selection of bacterial strains to formulate the consortium

According to the results of Tables 1 and 2, several strains could be chosen to design a formulation containing several bacterial species. In this work, three selection criteria were used to formulate the bacterial consortium: ability to coexist, resistance to desiccation and potential to promote plant growth as supported by the literature [9,51]. Therefore, four compatible strains were selected to formulate the bacterial consortium; three that are highly tolerant to desiccation (*A*. *brasilense* Sp7, *Acinetobacter* sp. EMM02, and *Sphingomonas* sp. OF178) and one that is moderately tolerant (*P*. *putida* KT2440). Despite its moderate desiccation tolerance, *P*. *putida* KT2440 was included in the formulation because of its well-known biotechnological and agronomic potential [8,9,51,52].

It is important highlight that *Acinetobacter* sp. EMM02 was isolated and characterized for the first time in this work (S1 Fig and S2 Table). This strain was partially characterized through the amplification and sequencing of the gene encoding 16S rRNA. Sequence analysis allowed the identification of this strain as part of the genus *Acinetobacter* and being closely related to *Acinetobacter calcoaceticus* (> 98% identity, S2 Table) and *Acinetobacter rhizosphaerae* (98% identity, S2 Table).

### Compatibility of the strains selected for the bacterial consortium under different growing conditions

The composition of the culture medium can affect the production of inhibitory substances [53,54] and consequently the establishment of antagonistic relations among microorganisms [24,30]. The validation of the compatibility of the strains selected to formulate the bacterial consortium was performed using growth curves under different culture conditions to evaluate any inhibitory relationships. Thus, studies of antagonism were performed using a double agar layer with solid PY-Ca medium (Table 1), and assays in liquid SYP and PY-Ca media (S2 Fig). The four strains grew successfully in co-culture, showing that they were compatible independently of the culture medium conditions and suggesting that these strains could also be compatible in association with plants.

### Influence of soil type on the desiccation tolerance of the bacterial strains selected for the bacterial consortium

**Fig 1:**
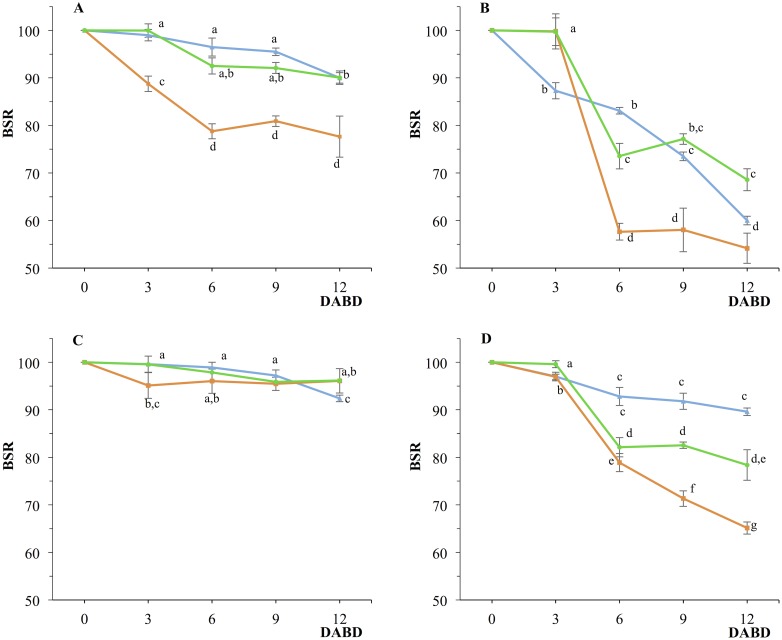
Bacterial survival ratio (BSR) response to desiccation stress using soils as support matrices for desiccation. A) *A*. *brasilense* Sp7, B) *P*. *putida* KT2440, C) *Acinetobacter* sp. EMM02, D) *Sphingomonas* sp. OF178. Green lines indicate desiccation using loamy sand for support, red lines indicates desiccation using sand for support, blue lines indicate bacterial desiccation without soil (control). Each point in each graph represents the mean of five independent determinations and the respective standard deviation. Values with identical letters in each graphic are not significantly different at p ≤ 0.05 based on Student’s *t* or Tukey’s test.

Bacteria-plant interactions are influenced by soil type [55,56] and likely the bacterial tolerance to desiccation as well. To determine whether soil type affects bacterial tolerance to desiccation, loamy sand and sand were used as substrates to evaluate the survival of the four bacterial strains in response to desiccation. The composition of these two substrates was analyzed using physicochemical assays (S3 Fig). Interestingly, *Acinetobacter* sp. EMM02 was highly resistant to desiccation independently of soil used (Fig 1). The survival of *A*. *brasilense* Sp7, *P*. *putida* KT2440 and *Sphingomonas* sp. OF178 in sand was lower than that in loamy sand or the water control, but a high number of bacteria was still found at the end of the desiccation experiment. The tolerance to desiccation of *A*. *brasilense* Sp7 and *P*. *putida* KT2440 in loamy soil was similar to that observed in the control (Fig 1). Despite a decrease in bacterial survival observed in the sandy soil for *A*. *brasilense* Sp7, *P*. *putida* KT2440 and *Sphingomonas* sp. OF178, it is worth mentioning that none of the bacteria in the formulation decreased to non-detectable numbers, which ensures the presence of bacteria even after desiccation stress.

### PGPR potential of the strains selected for the consortium

The four strains selected for the consortium showed high potential as PGPR (Table 3). They were able to produce siderophores (S4 Fig) and indole acetic acid (IAA) and perform phosphate solubilization (Table 3).

**Table 3:** Plant growth-promoting features of bacteria used for inoculant formulation.

Strain	Siderophores (halo mm)	Phosphates solubilization (halo mm)	Indole compounds (μg IAA/mg protein)
*Acinetobacter* sp. EMMS02	15.0 ±0.8	4.0 ±0.6	24.0 ±0.8
*Azospirillum brasilense* Sp7	10.0 ±0.5	1.0 ±0.5	32.7 ±1.5
*Pseudomonas putida* KT2440	20.0 ±0.5	4.0 ±0.7	8.0 ±0.6
*Sphingomonas* sp. OF-178A	15.0 ±0.4	3.0 ±1.2	4.0 ±0.8
Bacterial consortium	17.0 ±0.4	5.0 ±0.5	9.5 ±0.4

Values represent the media of five independent determinations and the respective standard deviation. *A*. *brasilense* Sp7 was used as the control strain. IAA (Indole Acetic Acid). *A*. *brasilense* Sp7 was used as the control strain.

*Azospirillum brasilense* Sp7 was used as control strain for determination of the PGPR characteristics, because it is known that this bacterium has the activities tested [57–59].

The mechanisms of plant growth promotion in bacteria closely related to the strains chosen in our work have been previously reported. For example, the production of IAA, a hormone for plant growth stimulation, has been described for *A*. *brasilense* [57], *Acinetobacter rhizosphaerae* Strain BIHB 723 [34] and *P*. *putida* GN04 [6]. Phosphate solubilization can be carried out by *A*. *brasilense* [59], *P*. *putida* GN04 [8] and *A*. *rhizosphaerae* Strain BIHB 723 [34]. Indirect mechanisms to promote plant growth, such as antagonism of pathogens, have also been reported for *P*. *putida* KT2440 [8] and *A*. *brasilense* [34]. Siderophore production has been reported for *P*. *putida* KT2440 [9,51] and *A*. *brasilense* Sp7 [60]. *P*. *putida* KT2440 may also antagonize the anthracnose fungus *Colletotrichum graminicola*, associated with maize plants, by triggering induced systemic resistance (ISR) [9].

Adhesion of bacteria to the seeds is the first step in PGPR-plant interactions [9,56], and the colonization of beneficial bacteria is fundamental to obtaining positive results in terms of the growth of plants caused by bacterial inoculation [9]. Desiccation stress in bacteria associated with seeds could influence the capability of bacteria to maintain adhesion or to carry out rhizospheric colonization. In the present work, the ability of bacteria (individually or in a bacterial consortium) to perform adhesion, colonization and plant growth promotion was evaluated under two experimental conditions before sowing the inoculated seeds: inoculated seeds without desiccation stress and inoculated and desiccated seeds.

### Bacterial inoculation of maize seeds (without desiccation stress) and its effect on plant growth (Exp. 1)

The number of bacteria was quantified for the consortium or individual bacterial suspensions used in this experiment, and the results are shown in S3 Table.

The four strains showed good adhesion to the seeds for both mono-inoculation and co-inoculation (bacterial consortium). The number of bacteria that adhered to maize ranged from 10^5^ to 10^7^ CFU/seed (S4 Table). The adhesion of *P*. *putida* KT2440 to blue maize seeds was similar to that observed for hybrid maize (var. Golden Jubilee, West Coast Seeds, Canada) [61].

*A*. *brasilense* Sp7, *Acinetobacter* sp. EMM02, *Sphingomonas* sp. OF178, and *P*. *putida* KT2440 also showed good colonization in the plant rhizosphere in both individual inoculations and the bacterial consortium. The presence of bacteria in the rhizosphere was detected during the development of the plants, as shown by the data from 45 days after sowing (DAS). Bacterial colonization of the plants inoculated with the bacterial consortium was similar to that observed for the mono-inoculated plants (S5 Table). The bacterial populations associated with the rhizosphere were observed in the range of 10^5^ to 10^8^ CFU/g vermiculite (V) for *A*. *brasilense* Sp7, *P*. *putida* KT2440, *Acinetobacter* sp. EMM02 and *Sphingomonas* sp. OF178 for both the single bacterial inoculations and the bacterial consortium inoculation.

The number of bacteria colonizing the rhizosphere has been reported for several bacteria, for example, *Burkholderia* sp., *B*. *megaterium* and *Sphingomonas* sp. colonize the rhizosphere of corn plants with counts of 10^4^ CFU/g fresh soil after 5 weeks of plant growth [61], and *P*. *fluorescens* colonizes corn roots at populations of 10^5^ CFU/g in the rhizosphere after five weeks of plant growth [62].

**Fig 2:**
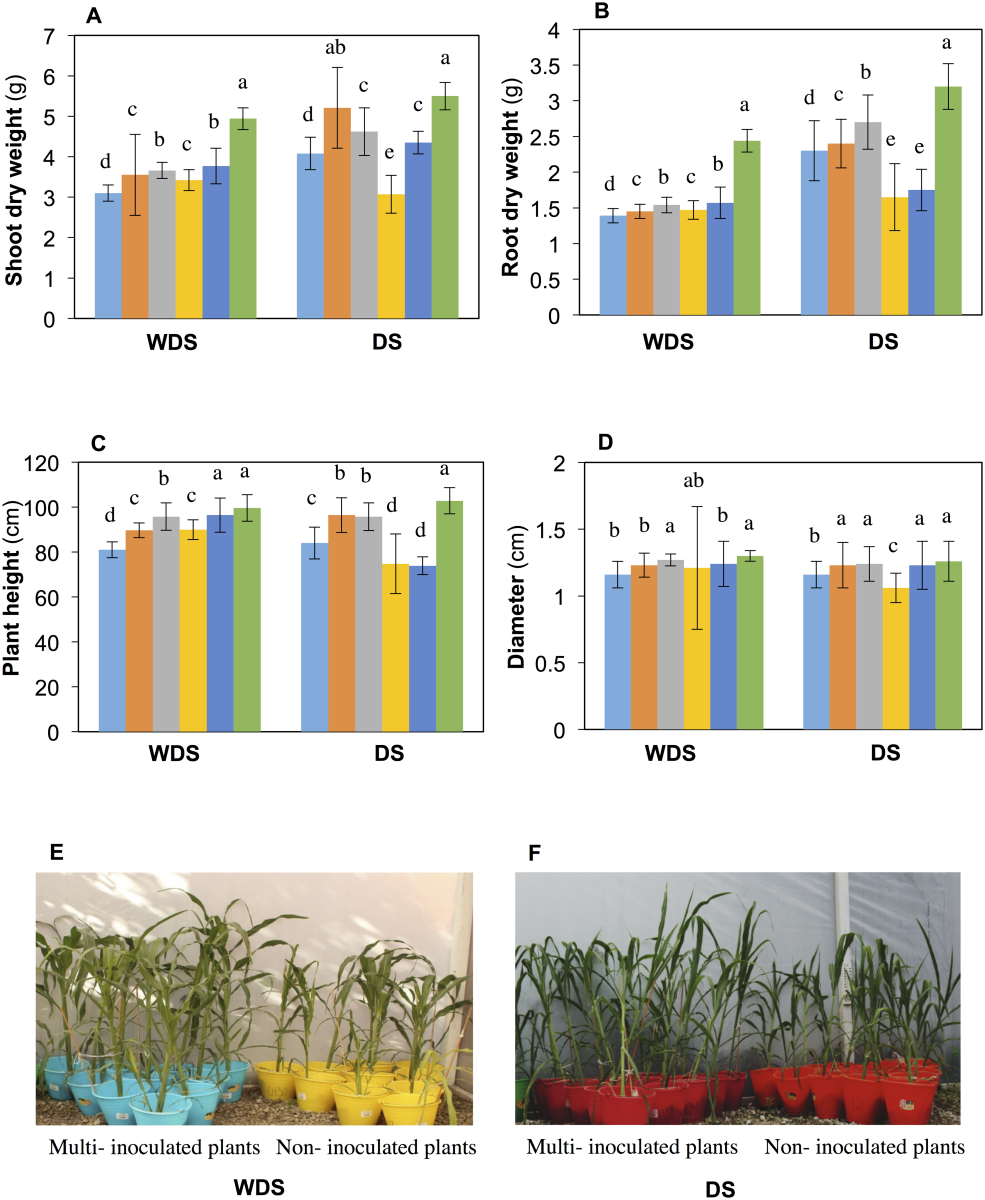
Effect of bacterial inoculation on the growth of maize 45 days after sowing (DAS) under greenhouse conditions. A) Shoot dry weight, B) Root dry weight, C) Plant height, D) Plant diameter. WDS indicates experiment with seeds not subjected to desiccation stress before germination (Exp. 1). DS indicates experiment with seeds subjected to desiccation stress before germination (Exp. 2), E) Plants from germinated seeds inoculated with the bacterial consortium (WDS), F) Plants from germinated seeds inoculated with the bacterial consortium (DS). Bar color key: sky blue, non-inoculated control; orange, *A*. *brasilense* Sp7; gray, *P*. *putida* KT2440; yellow, *Acinetobacter* sp. EMM02; dark blue, *Sphingomonas* sp. OF178; green/bacterial consortium. Each value represents the media of the data for 35 independent plants with the respective standard deviation. Identical letters in each bar group indicate that the values were not significantly different at P ≤ 0.05 based on Student’s *t* or Tukey’s test.

The effects of single and multi-inoculation on the growth of maize plants were evaluated at 45 DAS under greenhouse conditions. It was observed that plants inoculated with individual strains (*P*. *putida* KT2440, *A*. *brasilense* Sp7, *Acinetobacter* sp. EMM02 and *Sphingomonas* sp. OF178) significantly increased in height and shoot and root dry weight with respect to non-inoculated plants (Fig 2C, 2A and 2B). There were no differences in the diameter of mono-inoculated plants and non-inoculated controls, except in the case of *P*. *putida* KT2440 inoculation (Fig 2D). Plants inoculated with the bacterial consortium always showed higher values of growth parameters compared to mono-inoculated plants or control plants (Fig 2). The shoot dry weight of plants inoculated with the bacterial consortium was 75% higher than that of control plants. In the same way, root dry weight, plant height, and stem diameter were 59%, 22%, and 12% higher in plants inoculated with the bacterial consortium than in non-inoculated controls (Fig 2). In general, bacterial consortium inoculation showed greater plant growth promotion and enhanced the appearance of plants compared to the non-inoculated controls (Fig 2A–2E).

The interaction among bacterial strains in the consortium could have a synergistic effect and improve plant performance, as was previously reported [63,64]. For instance, the inoculation of sugarcane with a bacterial consortium of five diazotrophic strains (*G*. *diazotrophicus*, *Herbaspirillum seropedicae*, *Herbspirillum rubrisubalbicans*, *Azospirillum amazonense*, and *P*. *tropica*) increases stem production [42]. The inoculation of chickpea and wheat plants with bacterial consortiums promotes higher plant growth than mono-inoculation [11,61]. The inoculation of tomato seeds with two strains of *P*. *fluorescens* and an arbuscular mycorrhizal fungus synergistically increases tomato growth compared to individual inoculation with each microorganism [64]. Furthermore, maize inoculated with an arbuscular mycorrhizal fungus and a PGPR consortium showed increases in the mineral nutrient content in leaves and biomass production [13]. Our data are in agreement with those results; seed inoculation with a bacterial consortium improved the growth of native blue maize compared to mono-inoculated plants and non-inoculated plants.

### Desiccation of inoculated seeds and its effect on plant growth (Exp. 2)

**Fig 3:**
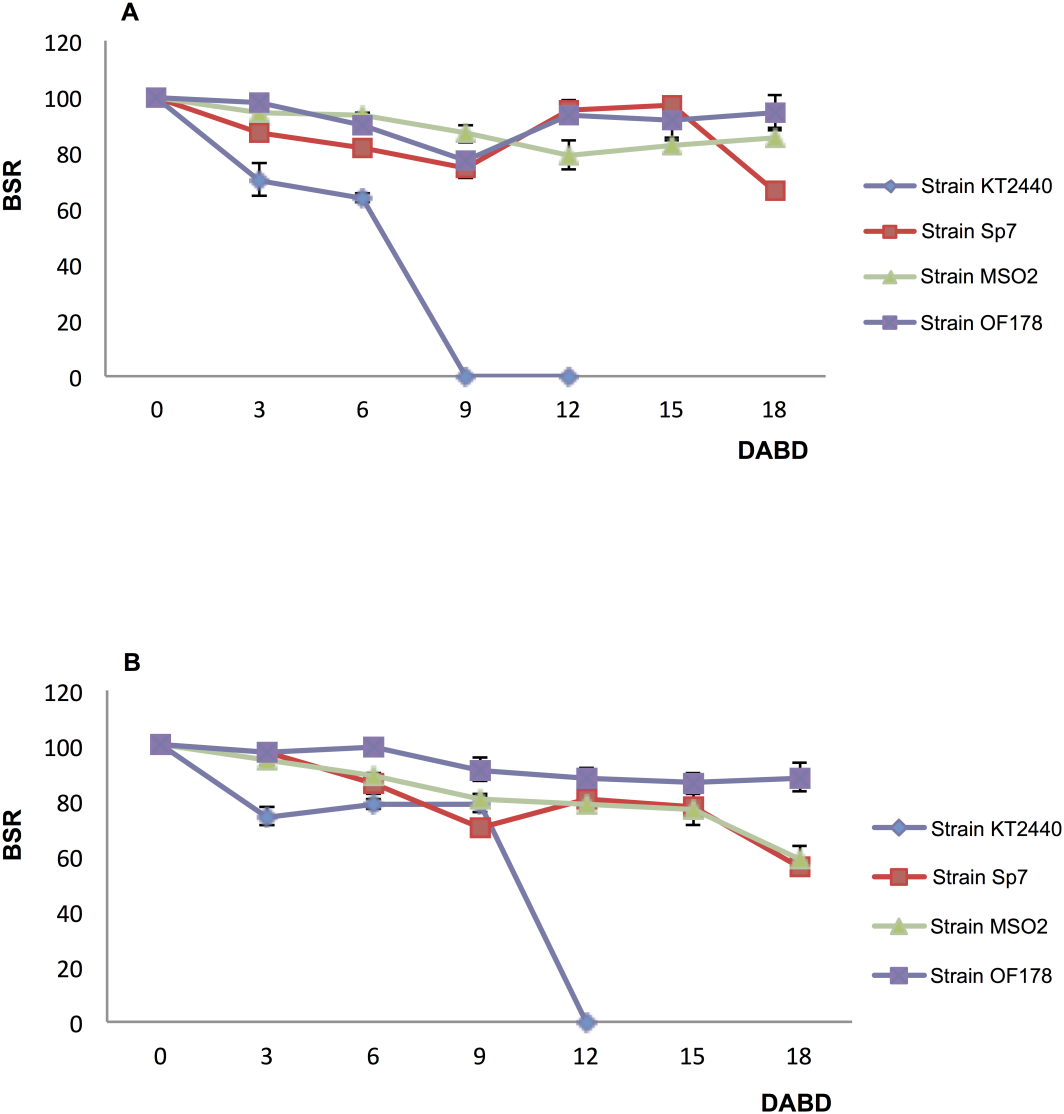
Bacterial survival ratio (BSR) of bacteria associated with blue maize seeds subjected to 18 days of desiccation. Fig 3A, BSR determination for seeds inoculated with single bacterial strains. Fig 3B, BSR determination for seeds inoculated with the bacterial consortium. Purple squares represent *Sphingomonas* sp. OF178, green triangles represent *Acinetobacter* sp. EMM02, red rectangles represent *A*. *brasilense* Sp7, and blue diamonds represent *P*. *putida* KT2440.

The number of bacteria of each strain present in the consortium or individual suspensions used in the second experiment is shown in S3 Table. Blue maize seeds were inoculated with single bacterium suspensions or the bacterial consortium and then placed into a desiccation chamber for eighteen days before being sown. The BSR of strains associated with seeds was calculated (Fig 3). In this experiment, *A*. *brasilense* Sp7, *Acinetobacter* sp. EMM02 and *Sphingomonas* sp. OF178 showed high tolerance to desiccation, and *P*. *putida* KT2440 was not detected after 12 DABD (days after the beginning of desiccation). For mono-inoculated seeds, the BSR values at 18 DABD were 66, 85, 94.5, and 0 for *A*. *brasilense* Sp7, *Acinetobacter* sp. EMM02, *Sphingomonas* sp. OF178 and *P*. *putida* KT2440, respectively (Fig 3A). Values of tolerance to desiccation of the seeds inoculated with the bacterial consortium at 18 DABD were 56, 59, 89 and 0, respectively (Fig 3B).

The survival of the three highly tolerant bacteria associated with seeds was similar in mono- and co-inoculations (Fig 3). In contrast, *P*. *putida* KT2440 in the co-inoculation showed higher tolerance (BSR of 78 at 9 DABD) than in the mono-inoculations (BSR of 0 at 9 DABD). Apparently, mixed inoculation allowed *P*. *putida* KT2440 to increase its tolerance to desiccation.

The adhesion of desiccated bacteria to seeds was evaluated 12 hours after sowing; it was observed that the three highly tolerant strains (*A*. *brasilense* Sp7, *Acinetobacter* sp. EMM02 and *Sphingomonas* sp. OF178) were present in high numbers for both the single and mixed inoculations (S4 Table). Under both tested conditions (Exp. 1 and 2), adhesion of bacteria was successful in acceptable numbers, except in the case of *P*. *putida* KT2440, which was not detected in Exp. 2.

Similar to Exp. 1, in Exp. 2, the rhizospheric colonization by the four strains showed high numbers in both the individual inoculations and multi-inoculation. The number of bacteria detected in the rhizosphere during plant development ranged from 10^5^ to 10^8^ CFU/g V (S5 Table). The bacterial colonization of plants inoculated with the bacterial consortium was similar to that observed for the mono-inoculated plants (S5 Table).

In Exp. 2, *P*. *putida* KT2440 was not detected on seeds after 12 DABD nor during adhesion following rehydration. Interestingly, this bacterium was detected colonizing the rhizosphere of plants in numbers of approximately 10^6^ CFU/g V at any of three stages of plant growth evaluated. Most likely, during the desiccation process, *P*. *putida* KT2440 goes into a viable but non-culturable (or non-cultivable) state and returns to a cultivable state after the interaction with the maize rhizosphere. This viable but non-culturable state has been reported for *P*. *putida* KT2440 in the Pasteurization process, suggesting that it can be returned to a cultivable state when environmental conditions do not cause stress to the microorganism, allowing it to maintain an optimal level of metabolic activity [65]. On the other hand, the metabolic capacity of *P*. *putida* F1 allows it to tolerate different stresses by entering metabolic states that are viable but not culturable (VBNC) and exhibiting near zero growth (NZG) [66].

The rhizospheric bacterial populations of the four strains evaluated in the present work were maintained throughout the durations of Exp.1 and Exp. 2, indicating that these bacteria can coexist without antagonistic effects in association with plants.

The identities of some rhizospheric strains isolated from the inoculated plants in Exp. 1 and 2 were corroborated. For this, the gene encoding 16S rRNA was amplified, and then a restriction pattern was generated with the restriction enzymes *Alu*I, *Hha*I, *Hinf*I, *Rsa*I, *Mbo*I and *Msp*I. Due to *Msp*I showed the restriction pattern with higher resolution of bands (S5 Fig), this enzyme was used for the corroboration of patterns of rhizospheric isolated strains. The rhizospheric strains evaluated for identification showed identical restriction patterns to those observed for the initially inoculated reference strains.

The plants inoculated with single strains in Exp. 2 showed a higher shoot dry weight than control plants at 45 DAS, except for *Acinetobacter* sp. EMM02 (Fig 2A). Compared to non-inoculated control plants, only mono-inoculation with *A*. *brasilense* Sp7 or *P*. *putida* KT2440 resulted in a higher root dry weight (Fig 2B). These results contrast with those for Exp. 1 (without desiccation stress), in which all strains inoculated individually were able to promote the growth of plants.

In Exp. 2, plants inoculated with the bacterial consortium showed a greater plant height and shoot and root dry weight than non-inoculated or mono-inoculated plants (Fig 2A–2C and 2F). The diameter of plants inoculated with the consortium was 8% higher than that of control plants. Plants inoculated with the bacterial consortium generally showed higher values of growth parameters than mono-inoculated plants, except for diameter (Fig 2D). Although the bacterial consortium underwent a desiccation process, it maintained its ability to promote the growth of maize after rehydration, showing its potential to increase plant growth, even under low water availability.

Other consortiums of bacteria that are resistant to desiccation also promote the growth of plants, for example, a consortium for maize composed of *Pseudomonas entomophila* GAP-P13, *Pseudomonas stutzeri* GRFHAP-P14, *P*. *putida* GAP-P45, *Pseudomonas syringae* GRFHYTP52 and *Pseudomonas monteilii* WAPP53 [46] and a consortium for pepper composed of *Microbacterium* sp. 3J1 and *Arthrobacter koreensis* 5J12A [46].

This work is one of the few studies showing the synergistic effects of a bacterial consortium on maize in terms of the promotion of plant growth and is the first showing the stimulation of native blue maize CAP15-1 TLAX. In our laboratory, other bacterial consortiums have been formulated with 5 or 6 bacterial strains that are able to coexist and have the ability to tolerate desiccation. Even though those formulations increase the growth of other plants, they do not increase the growth of blue maize plants, highlighting the importance of the bacterial consortium formulated with *P*. *putida* KT2440, *A*. *brasilense* Sp7, *Acinetobacter* sp. EMM02, and *Sphingomonas* sp. OF178.

Further studies will be necessary to determine which mechanisms are involved in the promotion of plant growth when these bacteria are interacting in the rhizosphere with native blue maize [67]. However, it is worth mentioning that *in vitro* assays of these four selected strains were positive for the production of indoles and siderophores and the solubilization of phosphates. These characteristics only suggest that all four strains could promote plant growth using these mechanisms.

## Conclusion

The inoculation of seeds of native blue maize with a bacterial consortium containing *P*. *putida* KT2440, *A*. *brasilense* Sp7, *Acinetobacter* sp. EMM02, and *Sphingomonas* sp. OF178 improved plant growth more effectively than mono-inoculation. After the bacterial consortium undergoes a desiccation process, it maintains its ability to promote blue maize growth after rehydration, showing the potential for using this bacterial consortium to increase plant growth, even under low water availability before germination. Future efforts will be necessary to determine which mechanisms are responsible for growth promotion when the bacteria in the consortiums are associated with plants. Finally, this bacterial consortium presents desirable traits for future application in blue maize cultivation and perhaps that of other native varieties, contributing to sustainable agricultural practices.

## Supporting information

S1 Fig Flow chart illustrating the identification of *Acinetobacter* sp. EMM02 by the amplification and sequencing of the gene encoding 16S rRNA. (PDF)

S2 Fig Bacterial growth in SYP or PY-Ca liquid media for bacteria grown individually or all together. (PDF)

S3 Fig Physicochemical characteristics of the soils used for support in the desiccation experiments. (PDF)

S4 Fig Siderophore production of the four strains chosen for the bacterial consortium. (PDF)

S5 Fig Restriction patterns of the amplified gene encoding the 16S rRNA of the bacterial strains isolated from plants. (PDF)

S1 Table Strains used in this study. (DOCX)

S2 Table Sequence identity of 16S rRNA from *Acinetobacter* sp. strain EMM02. (DOCX)

S3 Table Number of bacteria present in suspensions used for the inoculation of maize. (DOCX)

S4 Table Adhesion of strains to maize seeds sown in vermiculite. (DOCX)

S5 Table Colonization of the rhizosphere of maize plants by the bacterial strains. (DOCX)
