# Supplementary material for: Compatible bacterial mixture, tolerant to desiccation, improves maize plant growth
Source: PLoS One. 2017 Nov 8;12(11):e0187913. doi: 10.1371/journal.pone.0187913 (PMC5678714; doi:10.1371/journal.pone.0187913)
Supplement: S1 Fig — (PDF) [file pone.0187913.s001.pdf]

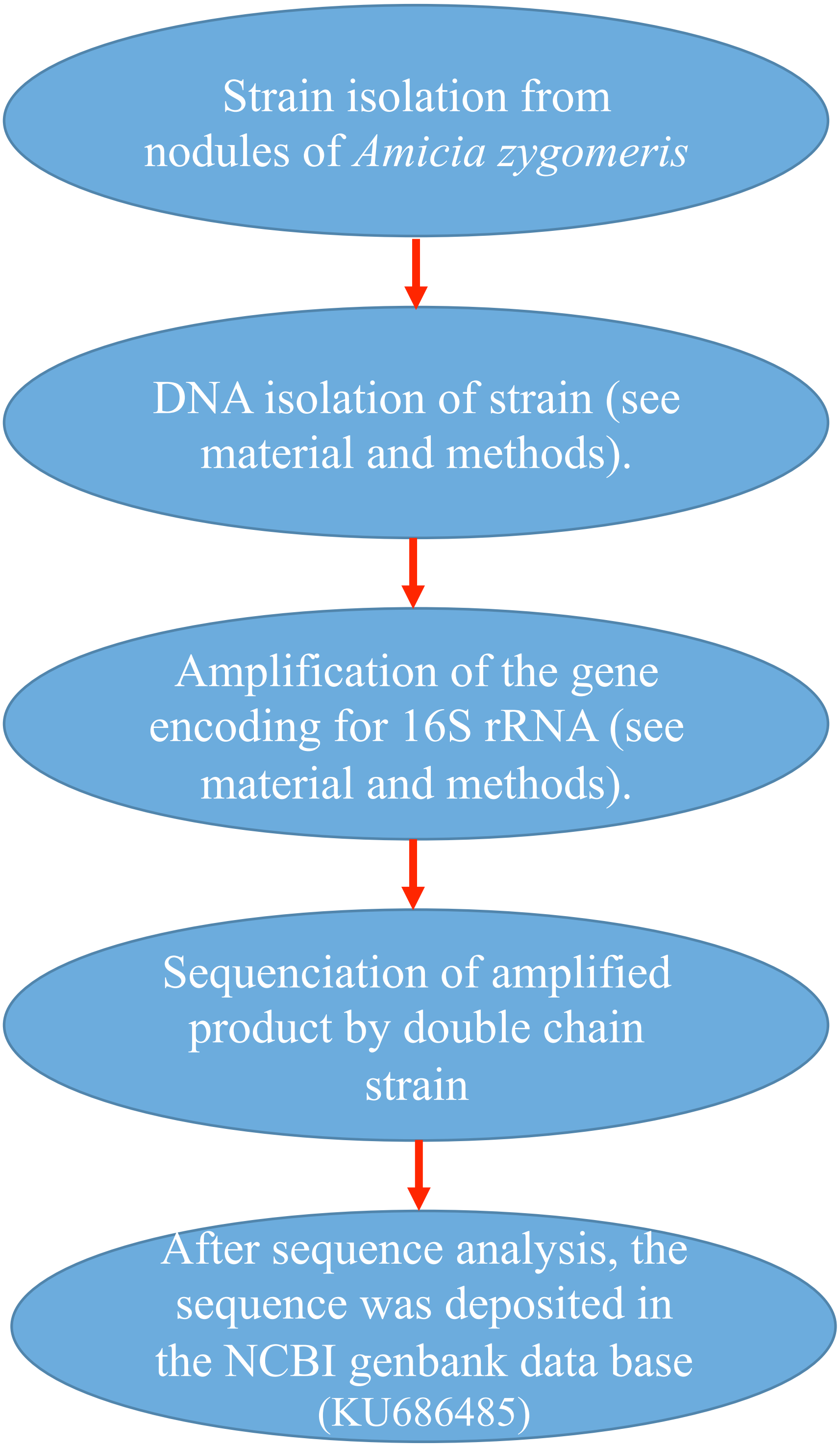

S1 Fig. Flow-chart to illustrate the identification of *Acinetobacter* sp. EMM02 by amplification and sequentiation of the gene encoding for 16S rRNA. The Genbank accession number is indicated in parenthesis.
