# Supplementary material for: Compatible bacterial mixture, tolerant to desiccation, improves maize plant growth
Source: PLoS One. 2017 Nov 8;12(11):e0187913. doi: 10.1371/journal.pone.0187913 (PMC5678714; doi:10.1371/journal.pone.0187913)
Supplement: S2 Fig — (PDF) [file pone.0187913.s002.pdf]

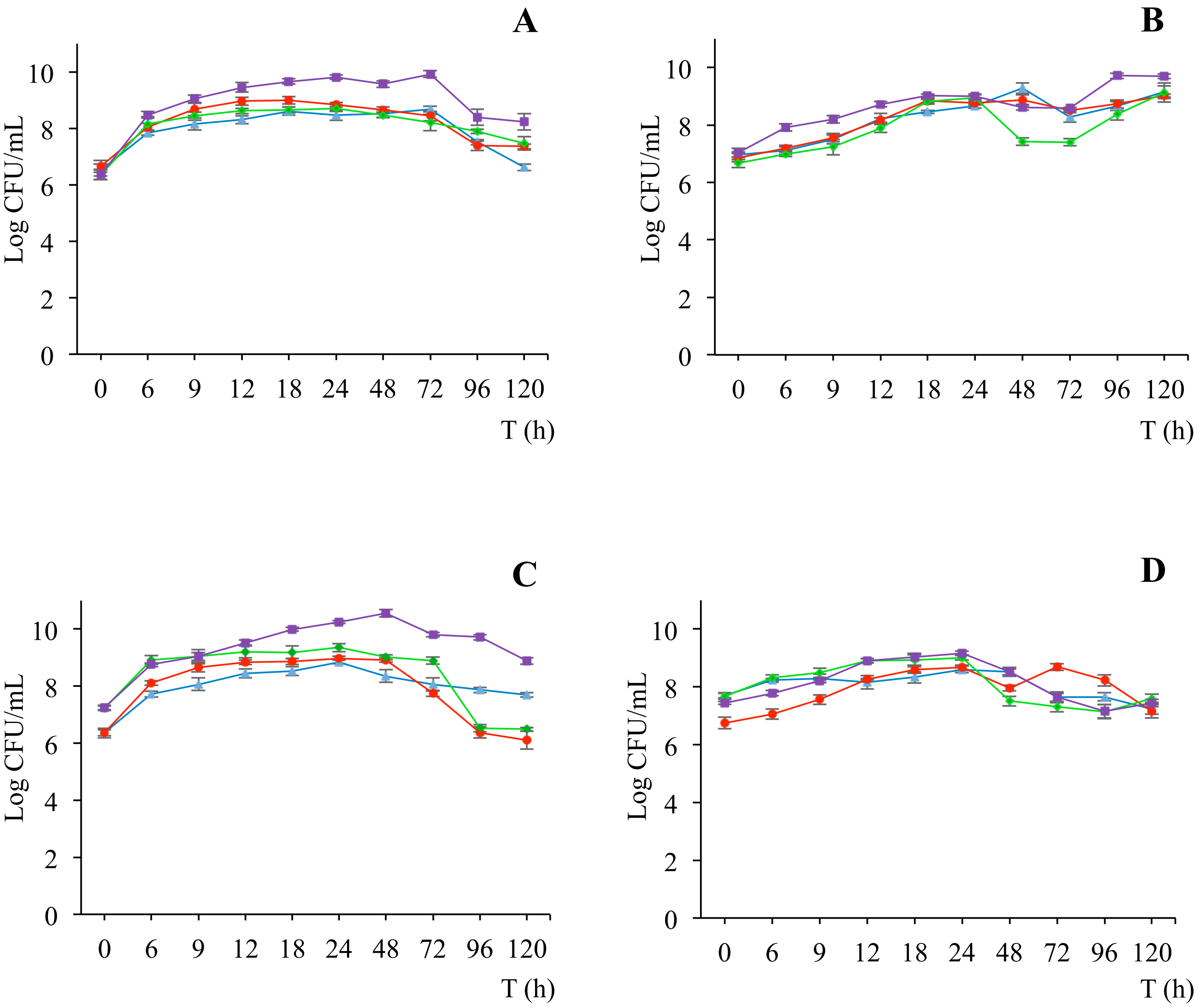

S2 Fig. Baterial growth in SYP or PY-Ca liquid media for bacteria grown individually or all together. A) Growth of the four strains individually inoculated in SYP liquid medium. B) Growth of the four strains under co-inoculation in SYP liquid medium. C) Growth of the four strains individually inoculated in PY-Ca liquid medium. D) Growth of the four strains under co-inoculation in PY-Ca liquid medium. Purple lines means the growth of *Sphingomonas* sp. OF178, red lines means the growth of *P. putida* KT2440, green lines means the growth of *Acinetobacter* sp. EMM02, and blue lines means the growth of *A. brasilense* Sp7. Each point represent the media of five independent determinations and the respective standard deviation. The growth of strains was similar both growing individually or together as a consortium.
