# Supplementary material for: Compatible bacterial mixture, tolerant to desiccation, improves maize plant growth
Source: PLoS One. 2017 Nov 8;12(11):e0187913. doi: 10.1371/journal.pone.0187913 (PMC5678714; doi:10.1371/journal.pone.0187913)
Supplement: S3 Fig — (PDF) [file pone.0187913.s003.pdf]

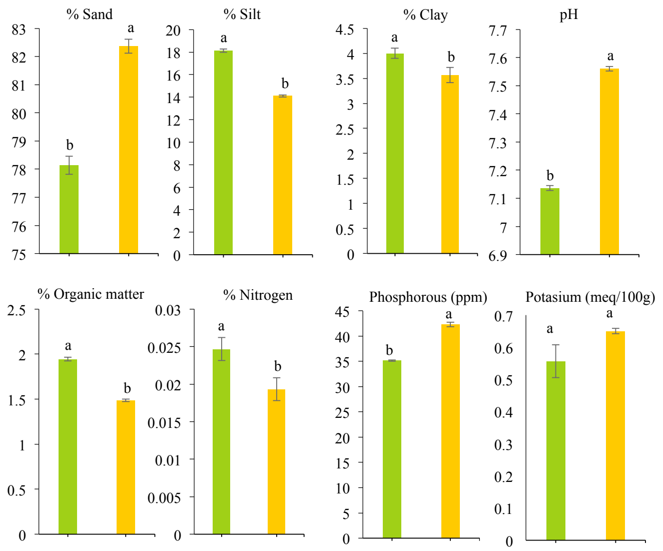

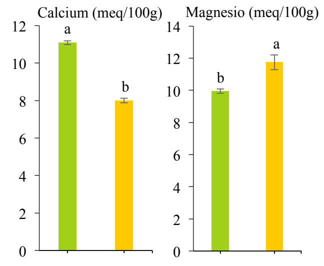

S3 Fig. Physicochemical characteristics of soils used as support for desiccation experiments. Each value of the bars represent the media of three determinations. Different letters in each graphic means that values of the bars differ statistically using *t*-student test to P≤0.05. Green bars represent the physicochemical characteristics of loamy sand soil and the orange the sand soil. The loamy sand soil was collected from Huejotzingo, Puebla, Mexico (19º 06’ 00´´ N; 98º 20’ 00’’ W) and the sand soil was collected from Domingo Arenas, Puebla, Mexico (19º 09’ 00’’ N; 98º 96’ 00’’ O).
