# Supplementary material for: Compatible bacterial mixture, tolerant to desiccation, improves maize plant growth
Source: PLoS One. 2017 Nov 8;12(11):e0187913. doi: 10.1371/journal.pone.0187913 (PMC5678714; doi:10.1371/journal.pone.0187913)
Supplement: S4 Fig — (PDF) [file pone.0187913.s004.pdf]

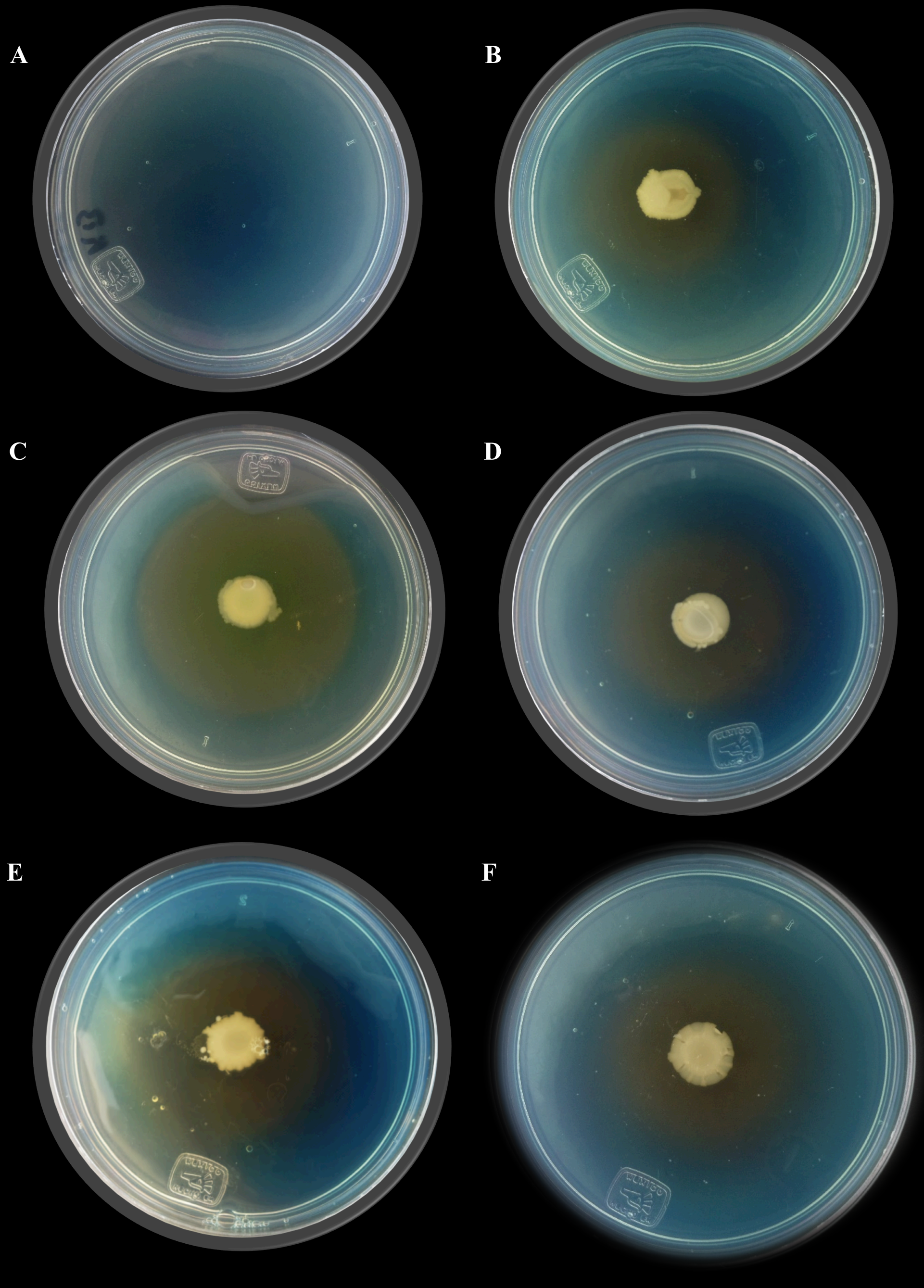

S4 Fig. Siderophore production of four strains chosen for the bacterial mixture. A) Control, B) *A. brasilense* sp. 7, C) *P. putida* KT2440, D) *Acinetobacter* sp. EMM02, E) *Sphingomonas* sp. OF178, and F) Bacterial mixture.
