# Supplementary material for: Compatible bacterial mixture, tolerant to desiccation, improves maize plant growth
Source: PLoS One. 2017 Nov 8;12(11):e0187913. doi: 10.1371/journal.pone.0187913 (PMC5678714; doi:10.1371/journal.pone.0187913)
Supplement: S5 Fig — (PDF) [file pone.0187913.s005.pdf]

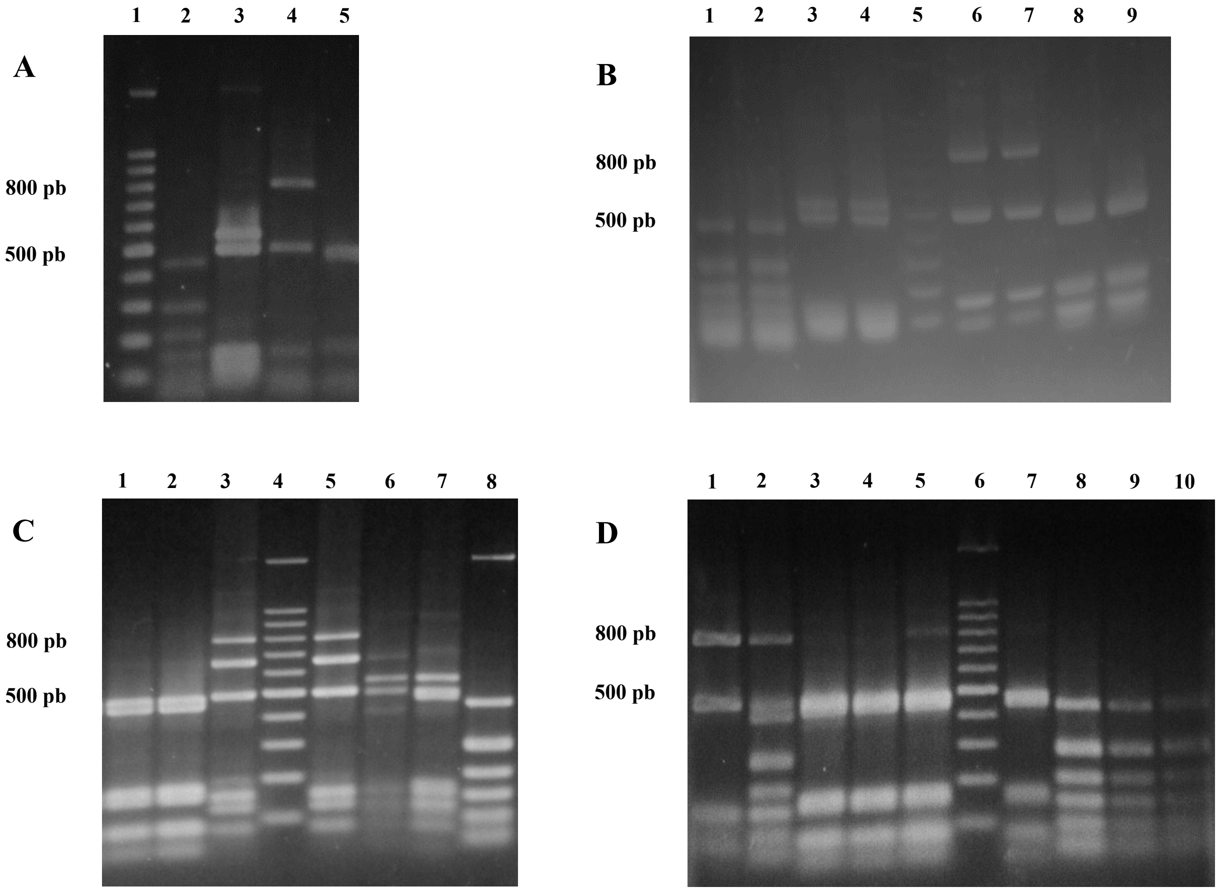

S5 Fig. Restriction patterns of amplified gene encoding for 16S rRNA of bacterial strains isolated from plants. (A) Restriction pattern of the amplified gene encoding for 16S rRNA using MspI of four reference strains used in the present work. 1) Molecular weight marker (100pb), 2) *A. brasilense* Sp7, 3) *P. putida* KT2440, 4) *Acinetobacter* sp. EMM02*,* 5) *Sphingomonas* sp. OF178. (B) Strains isolated from mono-inoculated plants (Exp. 1) and comparison with original strains. 1) Control *A. brasilense* Sp7. 2) Presumptive *A. brasilense* Sp7 strain-5 isolated on Selective Congo Red plates (SCRp). 3) Control *P. putida* KT2440. 4) Presumptive *P. putida* KT2440 strain-10 isolated on MM9- citrate plates (SMM9cp). 5) Molecular weight marker (100pb). 6) Control *Acinetobacter* sp. EMM02. 7) Presumptive *Acinetobacter* sp. EMM02 strain-20 isolated on Selective BAc plates (SBAcp). 7 Control *Sphingomonas* sp. OF178. 9) Presumptive *Sphingomonas* sp. OF178 strain-22 isolated on Selective LB 5% (SLBp).(C) Strains isolated from multi-inuculated plants (Exp. 1). 1) Presumptive *Sphingomonas* sp. OF178 strain-35 isolated on SLBp. 2) Presumptive *Sphingomonas* sp. OF178 strain-30 isolated on SLBp. 3) Presumptive *Acinetobacter* sp. EMM02 strain-46 and Presumptive *P. putida* KT2440 isolated on SBAcp. 4) Molecular weight marker (100pb). 5) Presumptive *Acinetobacter* sp. EMM02 strain-17 and Presumptive *P. putida* KT2440 isolated on SBAcp. 6) Presumptive *P. putida* KT2440 strain-20 isolated on SMM9cp. 7) Presumptive *P. putida* KT2440 strain-28 isolated on SMM9cp. 8) Presumptive *A. brasilense* Sp7 strain-42 isolated on (SRCcp). (D) Strains isolated from multi-inuculated plants (Exp. 2). 1) Presumptive *Acinetobacter* sp. EMM02 strain-41 isolated on SBAcp. 2) Non-purified isolated presumptive as *Acinetobacter* sp. EMM02 strain-38 isolated on SBAcp (Note: Pattern shows contamination with *A. brasilense* Sp7 strain). 3) Presumptive *Sphingomonas* sp. OF178 strain-49 isolated on SLBp. 4) Presumptive *Sphingomonas* sp. OF178 strain-21 isolated on SLBp. 5) Presumptive *Sphingomonas* sp. OF178 strain-18 isolated on SLBp. 6) Molecular weight marker (100pb). 7) Presumptive *Sphingomonas* sp. OF178 strain-19 isolated on SLBp. 8) Presumptive *A. brasilense* Sp7 strain-24 isolated on SCRp. 9) Presumptive *A. brasilense* Sp7 strain-28 isolated on SCRp. 10) Presumptive *A. brasilense* Sp7 strain-52 isolated on SCRp.
