# Supplementary material for: Compatible bacterial mixture, tolerant to desiccation, improves maize plant growth
Source: PLoS One. 2017 Nov 8;12(11):e0187913. doi: 10.1371/journal.pone.0187913 (PMC5678714; doi:10.1371/journal.pone.0187913)
Supplement: S1 Table — (DOCX) [file pone.0187913.s006.docx]

**S1 Table. Strains used in this study.**

| Strain | Source of isolation | Reference |
| --- | --- | --- |
| *Acinetobacter* sp*.* EMM02 | *Amicia zygomeris* | This work. GenBank accession number: KU686485; Fig S1 and S2 Table. |
| *Azospirillum brasilense* Sp7 | Grass rhizosphere | [1] |
| *Bradyrhizobium* sp. MS13 | Nodule of *Flemingia macrophylla* | Strain donated by Munive-Hernández A. (Collection strains from Molecular Microbial Ecology Laboratory, CICM-ICUAP) |
| *Bradyrhizobium* sp. MS21 | Nodule of *A. zygomeris* | Strain donated by Munive-Hernández A. (Collection strains from Molecular Microbial Ecology Laboratory, CICM-ICUAP) |
| *Bradyrhizobium* sp. MS22 | Nodule of *Trifolium repens* | Strain donated by Munive-Hernández A. (Collection strains from Molecular Microbial Ecology Laboratory, CICM-ICUAP) |
| *Bradyrhizobium* sp. MS23 | Nodule of *P. coccineus* | Strain donated by Munive-Hernández A. (Collection strains from Molecular Microbial Ecology Laboratory, CICM-ICUAP) |
| *Enterobacter* sp. UAPSO3001 | Maize | [5] |
| *Paraburkholderia tropica* MOc-725 | Maize | [2] |
| *Paraburkholderia tropica* MTo-293 | Maize | [2] |
| *Paraburkholderia tropica* MTo-672 | Maize | [3] |
| *Paraburkholderia tropica* Pp8^T^ | Sugarcane | [3,6] |
| *Paraburkholderia unamae* MTl-641^T^ | Maize | [2,6] |
| *Paraburkholderia unamae* ScCu23 | Sugarcane | [4] |
| *Pseudomonas putida* DOT-T1E | wastewater | [7] |
| *Pseudomonas putida* KT2440 | Soil | [8] |
| *Rhizobium* sp. MS23 | Nodule of *P. coccineus* | Strain donated by Munive-Hernández A. (Collection strains from Molecular Microbial Ecology Laboratory, CICM-ICUAP) |
| *Sphingomonas* sp. DS-201 | Bulk Soil | Strain donated by Boltner D. (Collection strains from Department of Environmental Protection, EEZ-CSIC) |
| *Sphingomonas* sp. DS-204 | Bulk soil | [9] |
| *Sphingomonas* sp. GOF-203 | Rhizosphere soil from  grasses | [9] |
| *Sphingomonas* sp*.* OF-178A | Bulk soil | [9] |

**S1 Table References**

1. Terrand, J., Krieg, N.R., and Dobereiner J. A taxonomic study of the Spirillum lipoferum group, with descriptions of a new genus, Azospirillum gen. nov. and two species, Azospirillum lipoferum (Beijerinck) comb. nov. and Azospirillum brasilense sp. nov. Can J Microbiol. 1978;24: 967–980.

2. Estrada-De Los Santos P, Bustillos-Cristales R, Caballero-Mellado J. Burkholderia, a Genus Rich in Plant-Associated Nitrogen Fixers with Wide Environmental and Geographic Distribution. Appl Environ Microbiol. 2001;67: 2790–2798. doi:10.1128/AEM.67.6.2790-2798.2001

3. Reis VM, Estrada-de los Santos P, Tenorio-Salgado S, Vogel J, Stoffels M, Guyon S, et al. Burkholderia tropica sp. nov., a novel nitrogen-fixing, plant-associated bacterium. Int J Syst Evol Microbiol. 2004;54: 2155–2162. doi:10.1099/ijs.0.02879-0

4. Caballero-Mellado J, Mart??nez-Aguilar L, Paredes-Valdez G, Estrada-de los Santos P. Burkholderia unamae sp. nov., an N2-fixing rhizospheric and endophytic species. Int J Syst Evol Microbiol. 2004;54: 1165–1172. doi:10.1099/ijs.0.02951-0

5. Morales YE, Juárez D, Aragón C, Mascarua M a, Bustillos MR, Fuentes LE, et al. Growth response of maize plantlets inoculated with Enterobacter spp., as a model for alternative agriculture. Rev Argent Microbiol. 2011;43: 287–293. doi:10.1590/S0325-75412011000400009

6. Sawana A, Adeolu M, Gupta RS. Molecular signatures and phylogenomic analysis of the genus burkholderia: Proposal for division of this genus into the emended genus burkholderia containing pathogenic organisms and a new genus paraburkholderia gen. nov. harboring environmental species. Front Genet. 2014;5: 1–22. doi:10.3389/fgene.2014.00429

7. Ramos-González MI, Godoy P, Alaminos M, Ben-Bassat A, Ramos JL. Physiological Characterization of Pseudomonas putida DOT-T1E Tolerance to p-Hydroxybenzoate. Appl Environ Microbiol. 2001;67: 4338–4341. doi:10.1128/AEM.67.9.4338-4341.2001

8. Nakazawa T. Travels of a Pseudomonas , from Japan. Environ Microbiol. 2002;4: 782–786.

9. Böltner D, Godoy P, Muñoz-Rojas J, Duque E, Moreno-Morillas S, Sánchez L, et al. Rhizoremediation of lindane by root-colonizing Sphingomonas. Microb Biotechnol. 2008;1: 87–93. doi:10.1111/j.1751-7915.2007.00004.x
