# Supplementary material for: Compatible bacterial mixture, tolerant to desiccation, improves maize plant growth
Source: PLoS One. 2017 Nov 8;12(11):e0187913. doi: 10.1371/journal.pone.0187913 (PMC5678714; doi:10.1371/journal.pone.0187913)
Supplement: S2 Table — (DOCX) [file pone.0187913.s007.docx]

**S2 Table.** **Sequence identity of 16S rRNA from *Acinetobacter* sp. strain EMM02.**

| **Strain** | **% identity** |
| --- | --- |
| *Acinetobacter* sp. EMM02 (KU686485**)** | 100 |
| *Acinetobacter calcoaceticus* (AY277552) | 99 |
| *Acinetobacter calcoaceticus* (FJ860876) | 98 |
| *Acinetobacter calcoaceticus* (JQ781506) | 99 |
| *Acinetobacter calcoaceticus* (JX133214) | 99 |
| *Acinetobacter calcoaceticus* (KC247688) | 99 |
| *Acinetobacter calcoaceticus* (KC758143) | 99 |
| *Acinetobacter calcoaceticus* (KF762557) | 98 |
| *Acinetobacter calcoaceticus* (KF923419) | 99 |
| *Acinetobacter calcoaceticus* (KF704066) | 99 |
| *Acinetobacter calcoaceticus* (KF704067) | 99 |
| *Acinetobacter calcoaceticus* (KJ767372) | 98 |
| *Acinetobacter calcoaceticus* (KJ939472) | 98 |
| *Acinetobacter calcoaceticus* (KM114920) | 99 |
| *Acinetobacter calcoaceticus* (KP762556) | 98 |
| *Acinetobacter calcoaceticus* (KP762541) | 98 |
| *Acinetobacter calcoaceticus* (KP762557) | 98 |
| *Acinetobacter calcoaceticus* (KP762560) | 98 |
| *Acinetobacter calcoaceticus* (KP762563) | 98 |
| *Acinetobacter calcoaceticus* (KT369903) | 99 |
| *Acinetobacter calcoaceticus* (KT369887) | 99 |
| *Acinetobacter calcoaceticus* (KT634059) | 99 |
| *Acinetobacter calcoaceticus* (KU350599) | 99 |
| *Acinetobacter calcoaceticus* (LN995703) | 98 |
| *Acinetobacter calcoaceticus* (NR 113343) | 98 |
| *Acinetobacter calcoaceticus* (NR 117619) | 98 |
| *Acinetobacter calcoaceticus* (NR119113) | 98 |
| *Acinetobacter rhizosphaerae* (AM921638) | 98 |
| *Acinetobacter rhizosphaerae* (DQ536511) | 98 |
| *Acinetobacter rhizosphaerae* (FN600413) | 98 |
| *Acinetobacter rhizosphaerae* (JQ435662) | 98 |

The accession number of each bacterial species is shown in parentheses.
