# Supplementary material for: Compatible bacterial mixture, tolerant to desiccation, improves maize plant growth
Source: PLoS One. 2017 Nov 8;12(11):e0187913. doi: 10.1371/journal.pone.0187913 (PMC5678714; doi:10.1371/journal.pone.0187913)
Supplement: S3 Table — (DOCX) [file pone.0187913.s008.docx]

**S3 Table. Bacterial number present in suspensions used for inoculation of maize.**

| **Strain** | **Selection medium** | ***Log CFU/ml of single bacterial suspension** | ***Log CFU/ml of mixed bacterial suspension** | ****Log CFU/ml of single bacterial suspension** | ****Log CFU/ml of mixed bacterial suspension** |
| --- | --- | --- | --- | --- | --- |
| *Acinetobacter* sp. EMM02 | BAc CTX^30^ | 8.50 ±0.39 | 7.00 ±0.41 | 7.60 ±0.35 | 7.00 ±0.21 |
| *Azospirillum brasilense* Sp7 | Congo red Cro^50^ | 7.70 ±0.42 | 7.10 ±0.51 | 8.00 ±0.30 | 7.50 ±0.37 |
| *Pseudomonas putida* KT2440 | MM9- citrate Cm^150^ | 5.90 ±0.36 | 5.10 ±0.38 | 5.70 ±0.32 | 5.00 ±0.31 |
| *Sphingomonas* sp. OF178 | LB 5% AK^50^ | 8.80 ±0.42 | 8.30 ±0.24 | 8.73 ±0.27 | 8.10 ±0.31 |

Cell number in the suspension (Log CFU/ml) was determined in quintupled by MSDP method in selective solid media. Values represent the media of five replicates with the respective standard deviation. Cro=Ceftriaxone 50 µg /ml, Cm=chloramphenicol 150 µg /ml, CTX=cefotaxime 30 µg /ml, AK= amikacin 50 µg /ml. *Experiment with seeds no subjected to desiccation stress (Exp.1). **Experiment with seeds subjected to desiccation stress (Exp.2).
