# Supplementary material for: Compatible bacterial mixture, tolerant to desiccation, improves maize plant growth
Source: PLoS One. 2017 Nov 8;12(11):e0187913. doi: 10.1371/journal.pone.0187913 (PMC5678714; doi:10.1371/journal.pone.0187913)
Supplement: S4 Table — (DOCX) [file pone.0187913.s009.docx]

**S4 Table. Adhesion of strains in maize seeds sowed in vermiculite.**

| **Treatment of inoculation** | ***Log CFU/seed** | **** Log CFU /seed** |
| --- | --- | --- |
| *Acinetobacter* sp. EMM02 | 7.87 ±0.20 | 7.19 ±0.21 |
| Bacterial mixture EMM02 | 7.48 ±0.35 | 6.70 ±0.3 |
| *Azospirillum brasilense* Sp7 | 5.71 ±0.41 | 4.63 ±0.46 |
| Bacterial mixture Sp7 | 7.68 ±0.18 | 4.25 ±0.30 |
| *Pseudomonas putida* KT2440 | 7.50 ±0.14 | 0 |
| Bacterial mixture KT2440 | 5.01 ±0.17 | 0 |
| *Sphingomonas* sp*.* OF178 | 6.55 ±0.33 | 7.35 ±0.36 |
| Bacterial mixture OF178 | 6.01 ±0.36 | 7.78 ±0.1 |

The bacterial population adhered to the seed surface was calculated using the MSPD method on selective media. Values represent the media of five replicates with the respective standard deviation. *Experiment with seeds no subjected to desiccation stress (Exp. 1). **Data for inoculated seeds that were subjected to desiccation for 18 days before planting in vermiculite (Exp. 2). Non inoculated seeds did not show any bacterial growth.*Inoculation of seeds for Exp. 1. **Seeds inoculation (Exp. 2).
