# Supplementary material for: Compatible bacterial mixture, tolerant to desiccation, improves maize plant growth
Source: PLoS One. 2017 Nov 8;12(11):e0187913. doi: 10.1371/journal.pone.0187913 (PMC5678714; doi:10.1371/journal.pone.0187913)
Supplement: S5 Table — (DOCX) [file pone.0187913.s010.docx]

**S5 Table. Colonization of the rhizosphere of maize plants by the bacterial strains.**

| **Treatment of inoculation** | ***15 DAS**  **Log CFU/g V** | ***30 DAS**  **Log CFU/g V** | ***45 DAS**  **Log CFU/g V** | ****15 DAS**  **Log CFU/g V** | ****30 DAS**  **Log CFU/g V** | ****45 DAS**  **Log CFU/g V** |
| --- | --- | --- | --- | --- | --- | --- |
| *Acinetobacter* sp. EMM02 | 6.70 ±0.14 | 6.50 ±0.34 | 7.60 ±0.25 | 5.60 ±0.22 | 6.10 ±0.36 | 7.60 ±0.31 |
| Bacterial consortium *Acinetobacter* sp. EMM02 | 7.80 ±0.52 | 6.20 ±0.56 | 7.90 ±0.52 | 6.00 ±0.18 | 6.00 ±0.42 | 8.80 ±0.23 |
| *Azospirillum brasilense* Sp7 | 6.10 ±0.16 | 7.20 ±0.45 | 8.60 ±0.38 | 7.90 ±0.28 | 8.40 ±0.56 | 8.20 ±0.56 |
| Bacterial consortium  *A. brasilense* Sp7 | 6.90 ±0.19 | 7.20 ±0.54 | 8.50 ±0.32 | 8.40 ±0.34 | 8.30 ±0.45 | 8.60 ±0.41 |
| *Pseudomonas putida* KT2440 | 6.40 ±0.2 | 5.69 ±0.39 | 6.60 ±0.62 | 6.40 ±0.2 | 6.11 ±0.69 | 6.50 ±0.4 |
| Bacterial consortium  *P. putida* KT2440 | 7.00 ±0.26 | 4.10 ±0.47 | 5.90 ±0.69 | 6.70 ±0.26 | 6.80 ±0.37 | 5.50 ±0.39 |
| *Sphingomonas* sp*.* OF178 | 6.18 ±0.71 | 6.70 ±0.67 | 8.00 ±0.40 | 8.20 ±0.45 | 8.80 ±0.09 | 8.60 ±0.14 |
| Bacterial consortium  *Sphingomonas* sp. OF178 | 7.72 ±0.15 | 6.20 ±0.54 | 7.40 ±0.45 | 8.00 ±0.29 | 8.10 ±0.32 | 8.10 ±0.44 |

Each value represents the media of data for five independent plants with the respective standard deviation. DAS: days after sowing. Bacteria with different morphology to that observed for reference strains were isolated from non-inoculated plants; however, the number of these bacteria was lower than 10^3^ CFU/g V. *Rhizospheric bacterial number of plants grown up from seeds no subjected to desiccation stress (Exp. 1). ** Data of plants grown up from seeds subjected to desiccation before planting them for germination (Exp. 2).
